# Computational Exploration of Structural Hypotheses for an Additional Sequence in a Mammalian Mitochondrial Protein

**DOI:** 10.1371/journal.pone.0021871

**Published:** 2011-07-11

**Authors:** Aymen S. Yassin, Rajendra K. Agrawal, Nilesh K. Banavali

**Affiliations:** 1 Laboratory of Cellular and Molecular Basis of Diseases, Division of Translational Medicine, Wadsworth Center, New York State Department of Health, Albany, New York, United States of America; 2 Department of Biomedical Sciences, School of Public Health, State University of New York at Albany, Albany, New York, United States of America; 3 Laboratory of Computational and Structural Biology, Division of Genetics, Wadsworth Center, New York State Department of Health, Albany, New York, United States of America; Université Joseph Fourier, France

## Abstract

**Background:**

Proteins involved in mammalian mitochondrial translation, when compared to analogous bacterial proteins, frequently have additional sequence regions whose structural or functional roles are not always clear. For example, an additional short insert sequence in the bovine mitochondrial initiation factor 2 (IF2_mt_) seems sufficient to fulfill the added role of eubacterial initiation factor IF1. Prior to our recent cryo-EM study that showed IF2_mt_ to structurally occupy both the IF1 and IF2 binding sites, the spatial separation of these sites, and the short length of the insert sequence, posed ambiguity in whether it could perform the role of IF1 through occupation of the IF1 binding site on the ribosome.

**Results:**

The present study probes how well computational structure prediction methods can *a priori* address hypothesized roles of such additional sequences by creating quasi-atomic models of IF2_mt_ using bacterial IF2 cryo-EM densities (that lack the insert sequences). How such initial IF2_mt_ predictions differ from the observed IF2_mt_ cryo-EM map and how they can be suitably improved using further sequence analysis and flexible fitting are analyzed.

**Conclusions:**

By hypothesizing that the insert sequence occupies the IF1 binding site, continuous IF2_mt_ models that occupy both the IF2 and IF1 binding sites can be predicted computationally. These models can be improved by flexible fitting into the IF2_mt_ cryo-EM map to get reasonable quasi-atomic IF2_mt_ models, but the exact orientation of the insert structure may not be reproduced. Specific eukaryotic insert sequence conservation characteristics can be used to predict alternate IF2_mt_ models that have minor secondary structure rearrangements but fewer unusually extended linker regions. Computational structure prediction methods can thus be combined with medium-resolution cryo-EM maps to explore structure-function hypotheses for additional sequence regions and to guide further biochemical experiments, especially in mammalian systems where high-resolution structures are difficult to determine.

## Introduction

Ribosomes have to interact with a variety of translation factors and ligands to accurately polymerize amino acids into a protein based on the mRNA codon sequence [1]. Mammalian mitochondrial ribosomes (mitoribosomes) are responsible for synthesis of 13 inner membrane proteins, which are essential components of complexes involved in oxidative phosphorylation and generation of cellular energy [2], [3]. Mitochondrial [4] and bacterial [5], [6] ribosomes differ significantly in overall size sizes of individual ribosomal subunits and the overall RNA to protein mass ratio [7], [8], [9]. Many mitochondrial ribosomal proteins have no homology with known bacterial ribosomal proteins, but even amongst those that do, many have additional sequence regions whose role is not clear [8], [10]. Methods to probe the structural and functional role of such additional sequence regions are therefore required.

There are only two initiation factors required for initiating protein translation in mitoribosomes (IF2_mt_ and IF3_mt_) [11] as compared to three initiation factors in bacteria (IF1, IF2, IF3) [12]. Translation initiation in bacteria requires the formation of the 30S initiation complex with the initiator fMet-tRNA (fMet-tRNA_i_^Met^) in the peptidyl-tRNA binding (P) site (see ref. [13]). To prevent binding of tRNA_i_^fMet^ to the aminoacyl-tRNA binding (A) site instead, IF1 occupies an overlapping binding position of A-site tRNA on the small (30S) subunit [14], [15], [16]. IF2 promotes initiator tRNA binding to the P site on the small subunit and facilitates association of the large (50S) subunit to form the 70S initiation complex [17]. IF3 stabilizes the 30S pre-initiation complex by preventing premature docking of the large subunit [18], [19].

**Figure 1:**
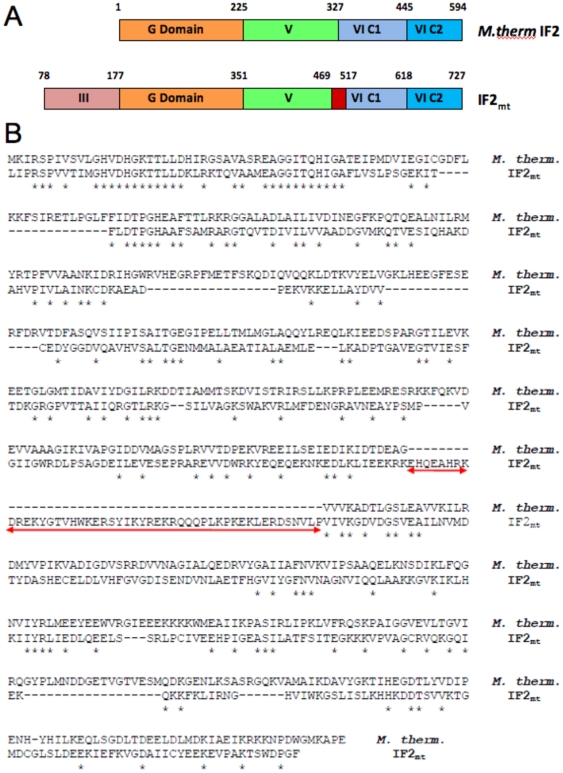
Domain architecture and sequence alignment of IF2_mt_ and *M. thermoautotrophicum* IF2. (A) Depiction of domain alignment with the IF2_mt_ insert region shown in red. (B) The manually adjusted ClustalW sequence alignment; the red arrow indicates the 49 aa insert sequence.

According to the *E. coli* nomenclature, IF2_mt_ is composed of four domains named: domain III, the G domain (or domain IV), domain V, and domain VI with two C-terminal sub-domains, C1 and C2, that are homologous to their bacterial counterparts [20] (**Figure 1A**). Sequence alignment of IF2_mt_ to *E. coli* IF2 indicates an insertion of 37 amino acid (aa) residues in IF2_mt_ between domains V and VI [21]. Mutations in this insertion domain reduce IF2_mt_ binding to the mitochondrial small ribosomal subunit and inhibit formation of the initiation complex [21]. Biochemical and genetic studies have indicated that IF2_mt_ can replace both bacterial IF1 and IF2 in an *E. coli* strain with IF1 and IF2 gene knockouts, but deletion of the 37 aa insertion from IF2_mt_ necessitates the presence of IF1 in *E. coli* [22]. This observation suggests that the 37 aa insertion in IF2_mt_ as compared to *E. coli* IF2 plays the same role as *E. coli* IF1.

Only one atomic resolution IF2 crystal structure for an archaeal IF2 from *Methanobacterium thermoautotrophicum (M. thermoautotrophicum)* is currently available [23]. Initiation complexes with bound IF2 have been studied at medium structural resolution using cryo-EM in two bacterial organisms, *E. coli* and *Thermus Thermophilus (T. thermophilus)* [24], [25], [26]. Interpretation of these cryo-EM maps has relied on building homology models for bound bacterial IF2 based on the archaeal IF2 crystal structure. It was clear from these maps that if the additional insert in IF2_mt_ bound the same region as the A-site tRNA, it had to be in a spatially distinct location as compared to the rest of IF2_mt_. Recently, a higher resolution cryo-EM map of IF2_mt_ bound to the *E. coli* ribosome showed that IF2_mt_ indeed occupies both the IF2 and IF1 binding sites [27], which also provides a reference against which the predicted models can be assessed and improved.

The archaeal IF2 crystal structure construct is composed of three domains (IV–VI, **Figure 1A**) [28]. Sequence alignment between IF2_mt_ and archaeal IF2 suggests the presence of an additional 49 aa residue sequence in IF2_mt_ between domains V and VI (**Figure 1B**). This is in contrast to the 37 aa residue insertion previously detected in IF2_mt_ when compared to *E. coli* IF2 [21]. In the present study, to investigate the structural feasibility for this insert region to occupy the same ribosomal binding site as IF1, a homology model for IF2_mt_ was created based on a sequence alignment of IF2_mt_ with archaeal IF2. The greater sequence homology of the domain VI-C1 and VI-C2 regions of IF2_mt_ to the bacterial C1 and C2 terminal domains from *Bacillus stearothermophilus* [29], [30], with available NMR structures, was also exploited to improve this homology model (also see ref. [27]). A protocol combining rigid body docking, flexible fitting, *ab initio* modeling of the insert region, its placement in the IF1 binding site, connection of this insert to the rest of the flexibly fit IF2_mt_, and final energetic optimization was used to generate two composite quasi-atomic models of IF2_mt_ bound to the *E. coli* and *T. thermophilus* ribosomes. The two models have the insert region occupying the IF1 binding site while the rest of IF2_mt_ occupies the separate IF2 binding site. These models already anticipate the structural feasibility of the 49 aa residue insert region to bind the spatially separated IF1 binding site, but cannot predict the binding orientation. Flexible fitting into the IF2_mt_ density map can provide a reasonable quasi-atomic model for the IF2_mt_ insert. Lack of conservation in the sequence neighboring the insert region can be used to obtain alternate models that have less extended linker regions. This study provides an example of how specific hypotheses about structure-function relationships of mammalian macromolecular complexes could be initially probed by combining computational modeling with cryo-EM maps of bacterial or mitochondrial complexes.

## Results

### Automated modeling

**Figure 2:**
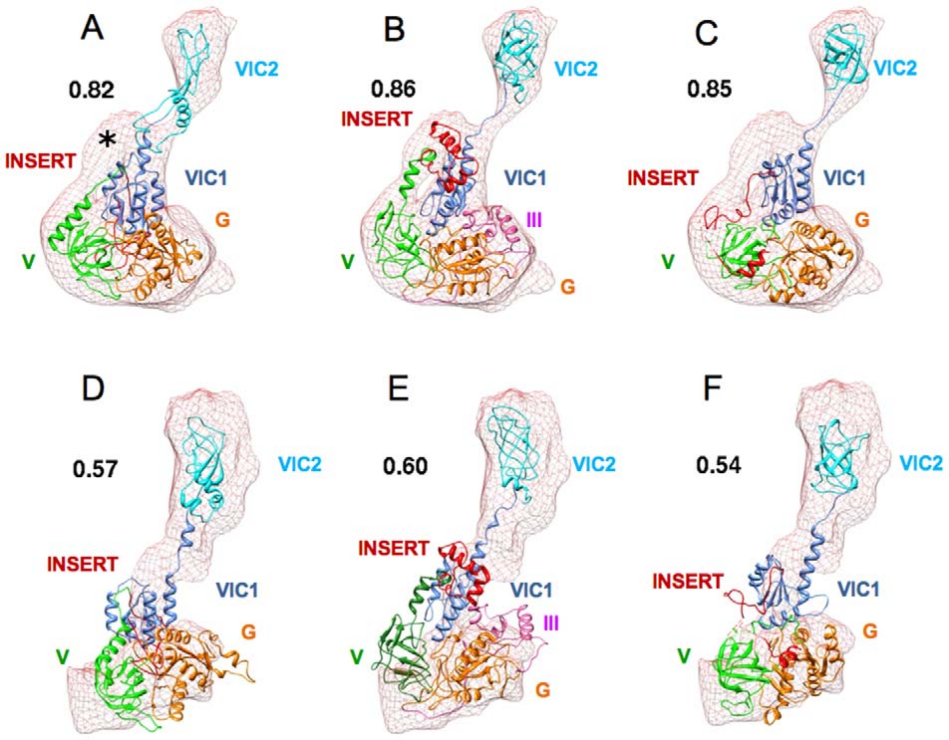
Flexible fits of automated complete IF2_mt_ models into IF2 cryo-EM maps. A–C represent flexible fits in excised map of *E. coli* IF2 [24]. D–F represent flexible fits in excised map of *T. thermophilus* IF2 [26]. A and D are automated MODELLER IF2_mt_ models, B and E are automated I-TASSER models, and C and F are automated SWISS-MODEL models. All models are flexibly fit using MDFF protocol 5 (see Methods section) and cross correlation coefficient (CCC) values of the fit are indicated next to each depicted model. The color scheme is as follows: domain G: orange, domain V: green, domain VI-C1: cornflower blue, domain VI-C2: cyan, and insert region: red. The black asterisk in panel A indicates the region of additional density originally assigned to the structurally unresolved N-terminal region of *E. coli* IF2 [24], and the higher CCC values in panels B and C are probably due to the enforced fit of the insert into this empty non-insert density.

Since IF2_mt_ shares a substantial sequence homology with archaeal IF2, for which the X-ray-crystallographic structure is known [23], it was necessary to first test the ability of automated homology modeling procedures to predict occupancy of both IF1 and IF2 binding sites by IF2_mt_. Homology models were generated by alignment between IF2_mt_ and archaeal IF2 sequences by utilizing the crystal structure of archaeal IF2. Two separate homology modeling procedures were used: Swiss Model Workspace [31], [32] and MODELLER [33]. An additional model was obtained using the automated *ab initio* I-TASSER protein structure prediction protocol [34]. The three models are different from one another, but domains G, V and VI in all three models seem well represented. The 49 aa insert in the first two models predicted by Swiss Model and MODELLER appears to be relatively unstructured, while I-TASSER predicts it to have some helical content. It is closely associated with domain V and the C1 sub-domain of domain VI in all three initial models. It is possible that any of these isolated models are correct, in that the 49 aa residue insert may not have a fully formed structure that is separated from the rest of IF2_mt_, except when bound to a ribosome. When these models are individually flexibly fit into the two excised bacterial IF2 densities (shown in **Figure 2**), the insert region tries to fit into the existing density, and does not spontaneously separate from the rest of IF2_mt_. Since the bacterial IF2 densities should have no density corresponding to the insert sequence, these automated flexibly fit models do not provide any anticipation of how the 49 aa insert could play the same role as IF1.

### Structure of the 49 residue IF2_mt_ insert

**Figure 3:**
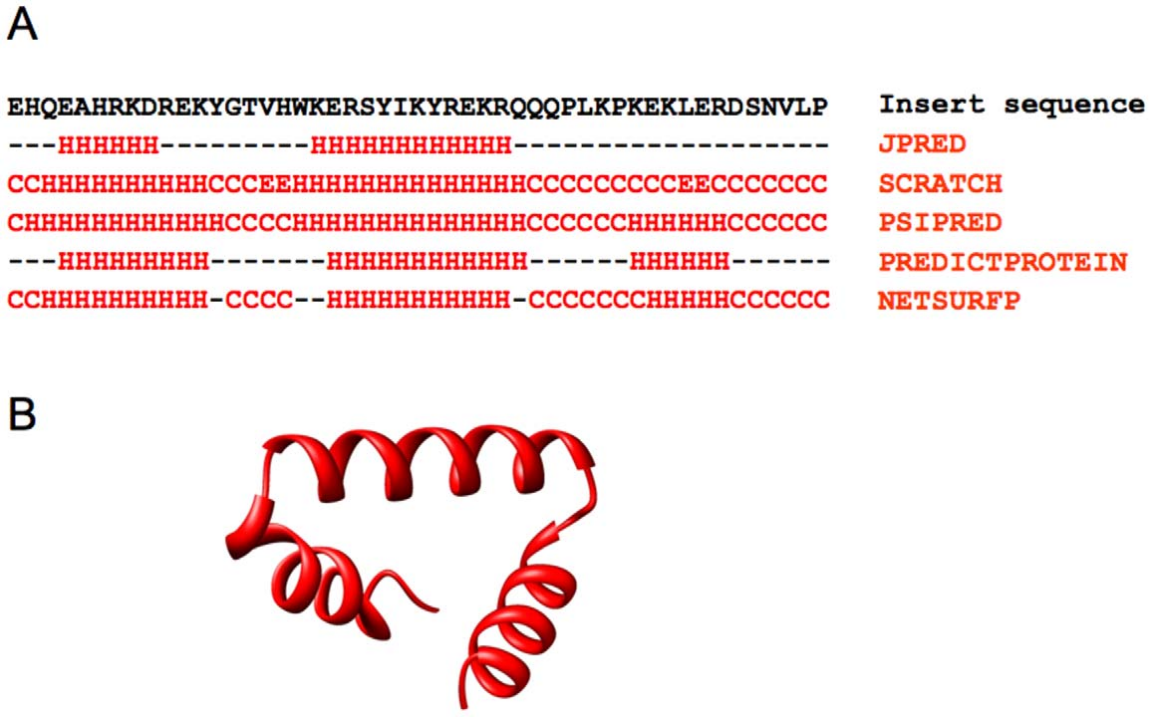
Secondary and tertiary structure prediction of the 49 aa insert region in IF2_mt_. (A) Five secondary structure prediction protocols: JPRED [35], SCRATCH [36], PSIPRED [37], PREDICTPROTEIN [38], and NETSURFP [39]; were used to predict the internal secondary structure of the 49 aa insert region in IF2_mt_. H represents α-Helix, C represents Coil, and E represents Extended strand. (B) Tertiary structure of the 49 aa insert as predicted by I-TASSER.

Since the 49 residue insert does not show any sequence homology to known crystal structures, multiple secondary structure prediction protocols [35], [36], [37], [38], [39] were used to characterize its internal secondary structure (**Figure 3A**). All secondary structure prediction protocols suggest that the 49 aa insert is at least partially helical. The two segments consistently predicted to be helical in all five protocols were the sequences EAHRKD and ERSYIKYREKR. These predictions suggest that the insert does not exactly structurally mimic bacterial IF1, which assumes a β-strand rich oligonucleotide binding (OB) fold both in isolation and in complex with the ribosome [15], [40]. This is not surprising given the shorter length of the insert as compared to IF1, which has more than 70 residues. However, there could be some similarity between the insert and the C terminal end of IF1, which contains α-helical structures that interact with the small ribosomal subunit [15], [40].

Since the structural prediction of the insert could be influenced by the presence of the other domains of IF2_mt_, an *ab initio* model of the insert by itself was generated using I-TASSER (**Figure 3B**). I-TASSER uses a hybrid protocol that incorporates secondary structure prediction methods [34] and is able to predict a helical structure for the 49 aa insert (also see ref. [27]). This model was used as the starting point to generate complete models of IF2_mt_ in which the ability of this 49 aa insert to extend to bind the same ribosomal binding site as IF1 could be assessed.

### Modeling and flexible fitting of IF2_mt_ into IF2 cryo-EM maps

**Figure 4:**
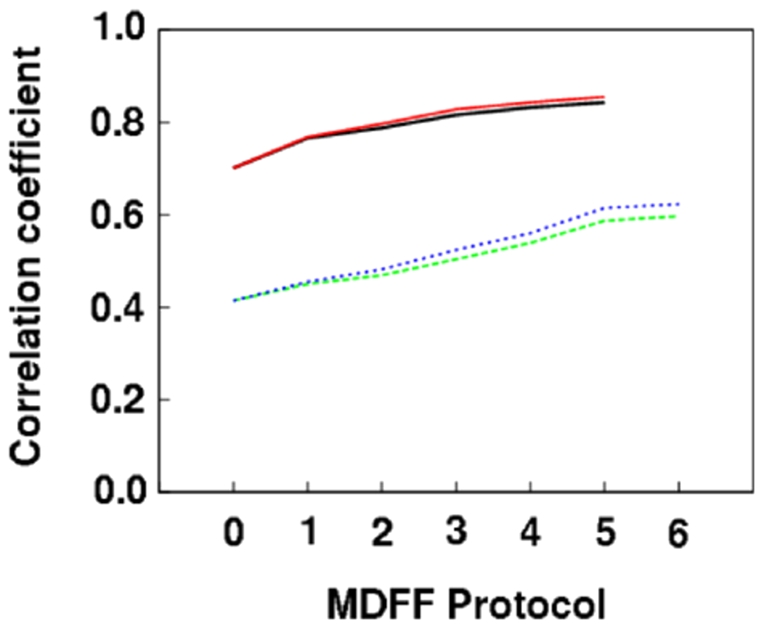
Optimization of flexible fitting MDFF protocols. If X is the number of dynamics steps and Y is the number of subsequent minimization steps, the six different MDFF protocols tested are: (1) X = 5000, Y = 700, (2) X = 10000, Y = 1200, (3) X = 20000, Y = 2200, (4) X = 50000, Y = 5200, (5) X = 100000, Y = 10200, (6) X = 150000, Y = 15200. MDFF protocol 0 represents the rigid-body docked initial models without any MDFF flexible fitting. The solid black line and the dotted green line represent cross correlation coefficients after X dynamics steps and only the first 200 minimization steps in the *E. coli* and *T. thermophilus* density maps, respectively. The solid red and dotted blue lines represent these values after all dynamics and minimization steps for the *E. coli* and *T. thermophilus* density maps, respectively.

The sequence homology of archaeal IF2 with IF2_mt_ is highest when the 49 residue insert sequence is excluded (**Figure 1**). There is also greater homology between domain VI-C1 and VI-C2 in IF2_mt_ and *B. stearothermophilus* as compared to the same regions in archaeal IF2. MODELLER [33] was used to generate a homology model of IF2_mt_ without insert by using manually adjusted optimal sequence alignments and the atomic resolution structures of archaeal IF2 and domains VI-C1 and VI-C2 in *B. stearothermophilus* IF2. Available cryo-EM reconstructions of IF2 bound to two bacterial ribosomes, *E. coli* [24] and *T. thermophilus* [26] were used. The homology model of IF2_mt_ (without its 49 aa insert) was flexibly fit into the corresponding regions of both cryo-EM maps. The flexible fitting was performed with Molecular Dynamics Flexible Fitting (MDFF) [41], [42] and the protocol was optimized to get the highest correlation coefficient, while minimizing over-fitting. The choice of MDFF protocol is illustrated in **Figure 4**. Correlation coefficients obtained with MDFF protocols varying in the numbers of dynamics and minimization steps and initial velocity distributions, show that MDFF protocol 5 utilizes the minimal number of steps to provide an optimal fit of the models to the two bacterial cryo-EM maps.

**Figure 5:**
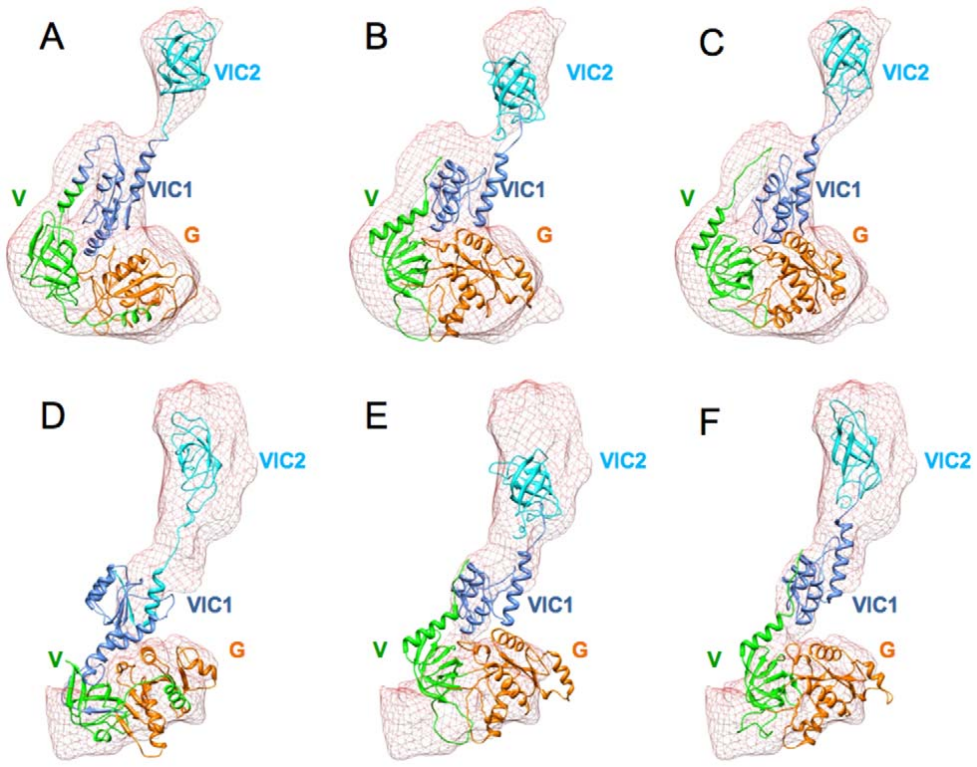
Flexible fitting of composite IF2_mt_ models without insert. A–C represent flexible fitting into the excised cryo-EM map of *E. coli* IF2 [24], D–F represent flexible fitting into the excised cryo-EM map of *T. thermophilus* IF2 [26]. (A) *E. coli* IF2 model from *Allen et al.* 2005 [24]. (B, E) Rigid body docked IF2_mt_ models without insert. (C, F) Flexibly fit IF2_mt_ models without insert. (D) *T. thermophilus* IF2 model (since this model is not deposited in the PDB, it was recreated using MODELLER based on the Simonettie *et al.* 2008 [26] sequence alignment and flexibly fit using MDFF protocol 5). The color scheme is the same as in Figure 2.

The next necessary step in obtaining reasonable models was flexible fitting of the initial IF2_mt_ model into the two IF2 cryo-EM maps. These flexible fits of the IF2_mt_ model are shown in **Figure 5**. The two flexibly fit IF2_mt_ structures are different from one another: the structure fit into the *E. coli* map appears more compact with lesser longitudinal separation of component domains. After flexible fitting with MDFF protocol 5, the cross correlation coefficients improved from 0.70 to 0.86 and from 0.41 to 0.62 for the *E. coli* and *T. thermophilus* maps, respectively. It should be noted that the cryo-EM maps do not have IF2_mt_ bound to either of these ribosomes, the density used for the fit corresponds to IF2 from the respective organisms. The better fit of IF2_mt_ (without its insert) into the *E. coli* map might be a low-resolution indication of greater structural similarity of IF2_mt_ to *E. coli* IF2 than to *T. thermophilus* IF2.

### Complete composite model of IF2_mt_

**Figure 6:**
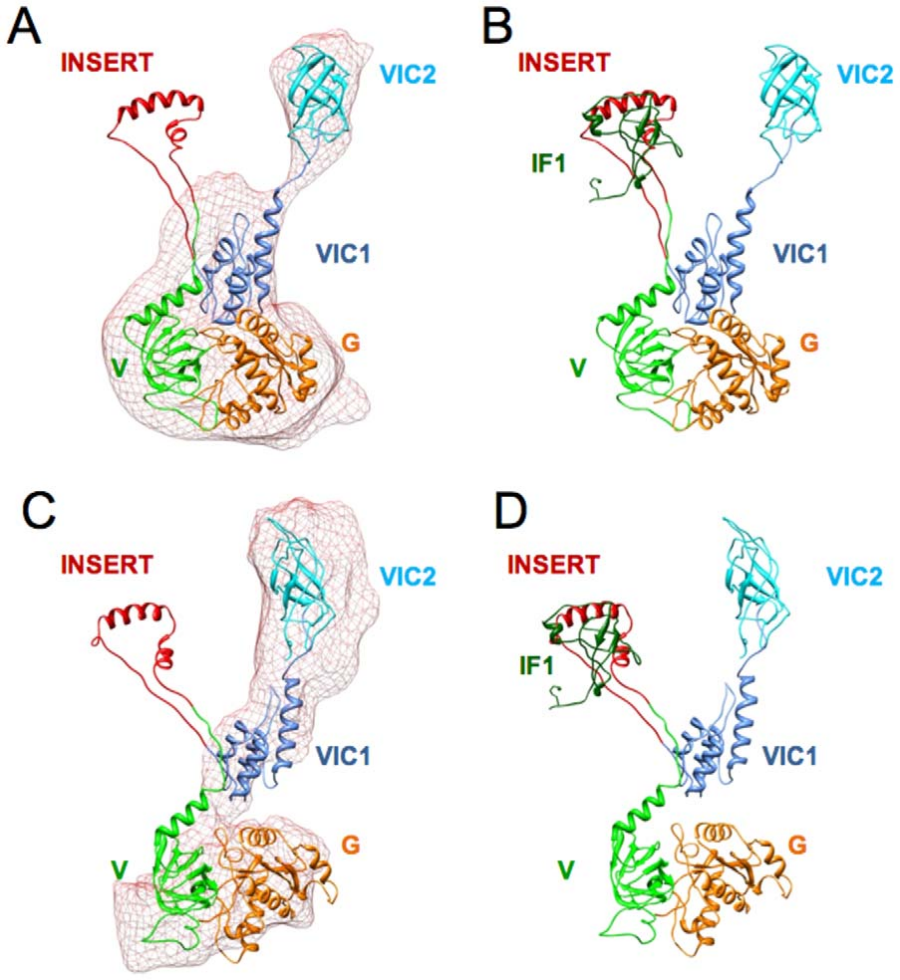
Final optimized composite IF2_mt_ models showing fit into excised IF2 cryo-EM densities and the structural overlay of the 49 aa insert with bacterial IF1. A–B represent the optimized IF2_mt_ model based on flexible fitting into the excised *E. coli* IF2 density map [24], C–D represent the optimized IF2_mt_ model based on flexible fitting into the excised *T. thermophilus* IF2 density map [26]. The insert is shown in red and the bacterial IF1 is shown in green. The position of IF1 is predicted based on the manual rigid-body fit of *T. thermophilus* 30S subunit with bound IF1 [15].

The last step in generation of a complete composite model of IF2_mt_ was to add the 49 aa residue insert region to the initial flexibly fit models of IF2_mt_ without insert. Since the hypothesis addressed by these models was whether the insert can occupy the same binding site as IF1, it was necessary to orient the insert in the same position as IF1. An automated prediction of the structural overlap using RAPIDO [43] did not yield a good structural overlap due to the sequence dissimilarity with *T. thermophilus* IF1, but a better overlap was obtained manually. The manually oriented insert was then connected to the rest of IF2_mt_ using LOOPY [44] for prediction of linker region structures. The complete model was then optimized using a series of restrained minimization and dynamics steps performed with CHARMM [45]. If the 49 aa insert region, which was absent in the maps, was also excluded from the correlation coefficient calculation, the fit of these models (shown in **Figures 6A and 6C**) were 0.83 and 0.61 for the *E. coli* and *T. thermophilus* maps, respectively.

The overlay of these models with IF1, shown in **Figure 6B and 6D**, illustrates that the predicted model has the insert extended into the IF1 binding site, while still maintaining some of its predicted helical secondary structure, and its covalent connection to the rest of IF2_mt_. It is also clear that such extension of the insert into the IF1 binding site would be much easier, and would maintain more of its original secondary structure, if some of its adjacent secondary structure elements were to refold in a more conducive orientation. However, in the absence of adjacent secondary structure element rearrangements, the primary structural adjustments required are the extended conformations assumed by the linkers at the edges of the 49 residue insert.

### IF2_mt_ binding to the ribosome

**Figure 7:**
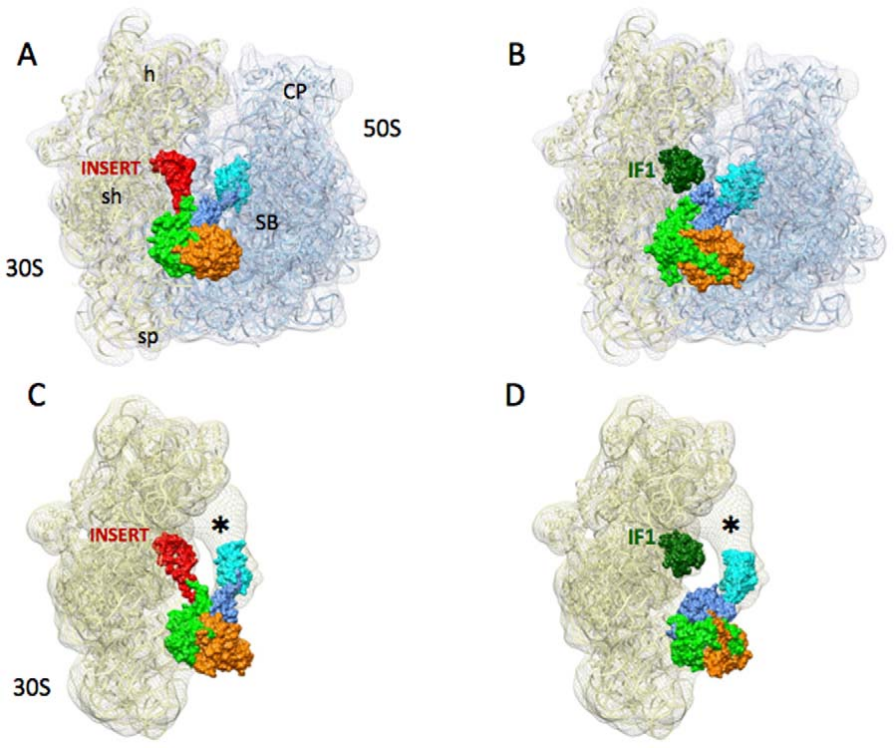
The occupation of *E. coli* and *T. thermophilus* IF1 and IF2 ribosomal binding sites by IF2_mt_. (A) Final optimized IF2_mt_ model bound to the *E. coli* 70S ribosome. (B) Bacterial IF1 and IF2 bound to the *E.coli* 70S ribosome, (C) IF2_mt_ model bound to the *T. thermophilus* 30S ribosomal subunit. (D) Bacterial IF1 and IF2 bound to the *T. thermophilus* 30S ribosomal subunit. Small subunit landmarks indicated: h - head, sh - shoulder, s - spur. Large subunit landmarks indicated: CP - central protuberance, SB - stalk base. The mesh density shown corresponds to the previously published *E. coli* 70S initiation complex (EMD 1248) [24] and the *T. thermophilus* 30S initiation complex (EMD 1523) [26]. In (C) and (D), the initiator tRNA density is indicated by an asterisk. The color scheme is as follows: domain G: orange, domain V: green, domain VI-C1: cornflower blue, domain VI-C2: cyan, and insert region: red, bacterial IF1 in dark green, small ribosomal subunit: transparent yellow, and large ribosomal subunit: transparent blue.

The optimized models are fit into excised IF2 maps, but their orientation inside this excised density affects their interactions with the other components of the translation initiation complex. **Figure 7** shows the two final optimized composite models of IF2_mt_ bound to the *E. coli* 70S ribosome and *T. thermophilus* 30S ribosomal subunit. As expected due to its partial fitting into the bound bacterial IF2 density, IF2_mt_ binds to the ribosome in the inter-subunit space. In both ribosomes, IF2_mt_ is in simultaneous contact with densities corresponding to the small subunit, the large subunit, as well as the initiator tRNA. The insert is engineered to occupy the position of the A-site tRNA on the small subunit, but does so without significantly disturbing the internal structure of the rest of IF2_mt_. These models address the structural feasibility of the IF1 and IF2 binding sites both being occupied by a single IF2_mt_ molecule. Without a high-resolution experimental density map of ribosome-bound IF2_mt_, it is not possible to be certain about the internal orientation of the insert region within the IF1 binding site. It is also not possible to exclude structural rearrangements in the internal structure of IF2_mt_ due to the presence of the insert.

### Eukaryotic IF2_mt_ insert sequence conservation

**Figure 8:**
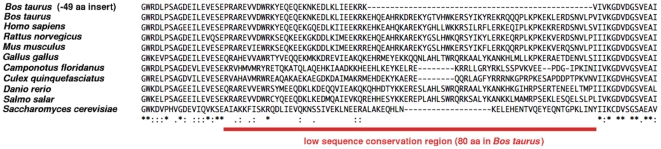
Sequence alignment of insert sequence region in representative eukaryotic IF2_mt_ sequences. Low sequence conservation region indicated by red bar, the first sequence shows the position of the 49 aa insert region as dashes.

The previous pair-wise sequence alignment of bovine IF2_mt_ with archaeal IF2 shown in **Figure 1** does not address the sequence variability of the insert region in the context of other eukaryotic IF2_mt_ sequences. To address this issue, a multiple sequence alignment of ten representative eukaryotic IF2_mt_ sequences in the vicinity of the insert sequence region was carried out (shown in **Figure 8**). The insert sequence is lodged between two regions of higher sequence conservation on its N- and C-terminal sides. However, the region of lower sequence conservation is greater on the N-terminal side than just the 49 residues identified based on comparison with the archaeal IF2_mt_ sequence. In *Bos taurus*, this region of low sequence conservation extends up to 80 aa residues. Within the smaller 49 aa region itself, there is variability even in the number of residues present, with the lowest number belonging to *Saccharomyces cerevisiae* (30 aa residues). If it is assumed that the lack of sequence conservation in this extended 80 aa region points to the possibility of a slightly altered secondary structure topological arrangement, it is possible to postulate alternate models for bovine IF2_mt_ that trade off minimizing the size of extended linker regions with adding a topological assumption of deviation from the crystallographically characterized topology for archaeal IF2.

### An alternate IF2_mt_ model

**Figure 9:**
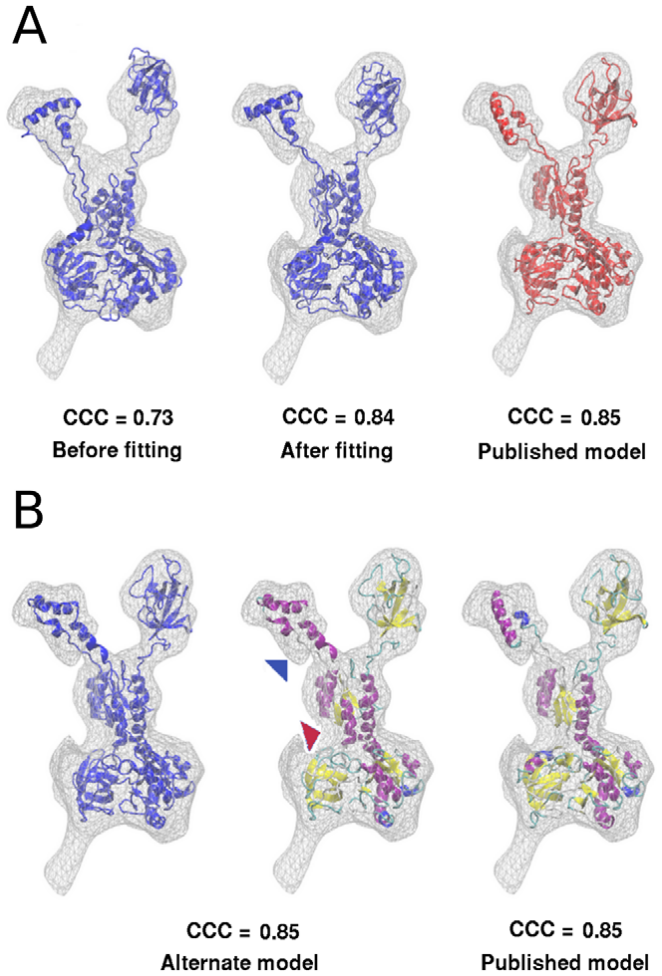
Computationally predicted quasi-atomic IF2_mt_ models and their flexible fitting into the IF2_mt_ cryo-EM map. (A) Comparison of IF2_mt_ model obtained by fitting into the *E. coli* IF2 cryo-EM map (in blue, left) with recently published IF2_mt_ model obtained by fitting into the IF2_mt_ cryo-EM map [27] (in red, right). The initial CCC value with the IF2_mt_ cryo-EM map is 0.73, but flexible fitting improves the CCC value to 0.84 (blue, center). (B) An alternate topologically variant model for IF2_mt_ (blue, left) and secondary structure differences between the alternate model (center) and the recently published IF2_mt_ model (right) [27]. The alternate model is obtained through reorientation of an α-helix (residues 446–460, in region indicated by red arrow) that results in additional helices being maintained in the insert region (indicated by a blue arrow). Color scheme for secondary structure: α-helices in purple, β-sheets in yellow, loops in cyan or white, and alternate helices in blue.

The higher cross correlation coefficient of the IF2_mt_ model predicted by fitting into the *E. coli* IF2 cryo-EM map [24] already suggests that it is likely to be a better model for IF2_mt_. If this model (blue model on left in **Figure 9A**) is structurally aligned to the published model of IF2_mt_ bound to the *E. coli* ribosome that was generated directly using the actual IF2_mt_ cryo-EM map (red model on right in **Figure 9A**) [27], there are many differences distributed throughout the molecule. The orientation of the two helices of the insert and the linkers connecting it to the rest of IF2_mt_ are especially different. Upon flexible fitting into the IF2_mt_ cryo-EM map (blue model in center in **Figure 9A**), the cross correlation coefficient improves from 0.73 to 0.84 and is only slightly lower that of the published model (0.85) [27]. However, the orientation of the insert region remains different, and the unusually extended linker regions are longer than the published model.

As stated before, if an added assumption of altered topology of secondary structure elements is applied, an alternate model can be created that has less extended linker regions. **Figure 9B** shows such an alternate model (left) and a secondary structure comparison between this alternate model (center) and the published IF2_mt_ model (right) [27]. This alternate model additionally assumes that a secondary structure element moves relative to the rest of the IF2_mt_ structure to accommodate the insert sequence. The change in orientation of a single α-helix (residues 446 to 460, region indicated by red arrow) results in greater maintenance of helical elements inside the insert sequence (region indicated by blue arrow) and a marked reduction in the presence of unusually extended linker regions. The two models do not vary in the objective measure of cross correlation coefficient of the fit in the cryo-EM density map (0.85 for both models) and thus equally fulfill the restraints provided by existing cryo-EM experimental data. Our published model [27] is still the preferred model simply through the Occam's razor principle, that is, it uses fewer speculative assumptions and fewer deviations from the only crystallographically known IF2 topology. Nevertheless, both these models can possibly be further distinguished through mutagenesis experiments that test the specific predictions of each model for interactions of the insert region with the *E. coli* ribosome.

## Discussion

Continuous internal connectivity of the IF2_mt_ models based on IF2 cryo-EM maps provides a basic *in silico* filter for the structural hypothesis that IF2_mt_ can play the dual role of bacterial IF1 and IF2 through occupation of the IF1 binding site by the insert sequence, which could be used to explain previous biochemical and genetic studies [22]. If continuous connectivity cannot be retained in predicted IF2_mt_ models where the insert occupies the IF1 site, this specific structural hypothesis can be inexpensively eliminated *in silico* prior to more definitive experimental studies. The assumptions in the construction of IF2_mt_ models preclude any predictions about whether the structure of IF2_mt_ in isolation, i.e., outside the context of the ribosome, would also have the insert region well separated from the rest of IF2_mt_. In other words, it is still possible that the insert region can play the role of IF1 by dynamically changing its orientation with respect to the rest of IF2_mt_ only in the presence of the ribosome, as has been observed previously in case of release factor 2 (RF2) [46].

The orientation of the secondary structure elements of the insert region itself can vary without significantly affecting the correlation coefficient of the overall fit to the IF2_mt_ density map [27]. Lack of sequence conservation among multiple eukaryotic IF2_mt_ insert regions also suggests the possibility of alternate models with slightly different secondary structure element orientations within this variable region. In one such alternate model, structural rearrangement of the orientation of a single α-helix allows for more insert sequence regions to be assigned helical secondary structures, again without reducing the correlation coefficient of the fit with the IF2_mt_ cryo-EM density. Such models involve a greater number of assumptions but provide experimentally falsifiable alternatives that can enhance the understanding of the structural and functional differences between mitochondrial and bacterial analogs.

Due to the greater difficulty of working on mammalian macromolecular complexes, there is significant value in usefully guiding experimental design using related structurally resolved bacterial macromolecular complexes by employing three dimensional computational modeling. The present approach can be utilized to generate multiple quasi-atomic models, invariant in their fit to medium resolution cryo-EM maps, but each with specific predictions for how insert regions interact with different complex components. These computational models, that each represent specific structural hypotheses, can then be used to guide experimental design for further refinement and validation through mutations, insertions, or deletions that are structurally predicted to modulate macromolecular function in distinguishable ways.

## Methods

### Sequence alignment and homology modeling

The cryo-EM density maps of the *E. coli* 70S initiation complex (EMD 1248) [24], the *T. thermophilus* 30S initiation complex (EMD 1523) [26], and IF2_mt_ bound to the *E. coli* ribosome [27] were used for generating the initial IF2_mt_ models. The crystal structure of the archaeal IF2 (PDB 1G7T) from *M. thermoautotrophicum* [23] was manually docked in corresponding IF2 electron densities in each map using Chimera [47]. The crystal structure of the 30S subunit from *T. Thermophilus* with bound IF1 (PDB 1HRO) [15] was similarly manually docked in the electron density map regions corresponding to the small subunits for both the *E. coli* and the *T. thermophilus* ribosomes to obtain the binding site and orientation of bacterial IF1 in both ribosomes. The crystal structure of the 70S *E. coli* ribosome [48] was also manually docked into the cryo-EM map of both *E. coli* translation initiation complexes.

Multiple sequence alignment of the representative eukaryotic IF2_mt_ sequences near the insert region were generated using ClustalW2 [49]. A pair-wise sequence alignment of the IF2_mt_ sequence with the archaeal IF2 sequence was also generated using ClustalW2 [49] and was manually adjusted to correct for any obvious misalignments. Since bovine IF2_mt_ is 727 aa [50] and archaeal IF2 is 594 aa, the first 175 aa residues in IF2_mt_, which represent the mitochondrial signal sequence (residues 1 to 77) and domain III (residues 78 to 175) that are absent in the archaeal IF2, were removed. In addition, by empirically removing additional residues on either side of the 37 aa insert region in IF2_mt_ as compared to *E. coli* IF2, it was observed that removing an additional three amino acids on the N-terminal side and nine amino acids on the C-terminal side of the 37 aa insert improved the sequence alignment between IF2_mt_ and archaeal IF2, yielding a slightly larger 49 aa insert region as compared to *E. coli* IF2. Alignment of the C1 and C2 sub-domains in domain VI of IF2_mt_ to their corresponding C1 (PDB 1Z9B, [29]) and C2 (PDB 1D1N, [30]) regions in the *B. stearothermophilus* IF2 sequence yielded higher sequence homology as compared to archaeal IF2. Removal of the initial 35 amino acids from the N-terminal end of the C1 sub-domain of *B. stearothermophilus* IF2 yielded the best sequence alignment.

These individual sequence alignments and the corresponding PDB files (1G7T,1Z9B and 1D1N) were used to build initial homology models for the respective bovine IF2_mt_ sequence regions using the program MODELLER [33]. The I-TASSER server [34] was used to build a separate *ab initio* model for the 49 aa insert region which was then manually aligned to the crystal structure of IF1 bound to the *T. thermophilus* 30S subunit [15] using the program VMD [51]. The models for the VI-C1 and VI-C2 sub-domains were structurally aligned to the corresponding regions in the overall homology model based on the archaeal crystal structure using the program RAPIDO [52]. A composite model of IF2_mt_ was then generated by connecting the 49 aa insert, and the C1 and C2 sub-domains based on *B. stearothermophilus* NMR structures [29], [30] to the rest of the IF2_mt_ modeled on the archaeal crystal structure using the program LOOPY [53] with the CHARMM22 protein parameters [54]. For linker structure prediction using LOOPY, only the minimal number of residues at each junction required to get the different domains covalently connected were randomized. To connect the 49 aa insert, VI-C1, and VI-C2 sub-domains to the main body of IF2_mt_, the minimal regions that required randomization and linker structure prediction with LOOPY were residues 464–473, 595–600, and 615–626, respectively. This initial IF2_mt_ model was then optimized using the program CHARMM [45] by multiple rounds of 5,000 steps of steepest descent (SD) and adopted basis newton raphson (ABNR) minimizations followed by 5,000 steps of room temperature langevin dynamics with a high friction coefficient (60 ps^−1^) in the presence of gradually reducing harmonic restraints on all non-hydrogen atoms. The insert and its neighboring regions (residues 464–518) were then subjected to similar rounds of minimization and dynamics under center-of-mass restraints to allow them to relax their internal structure, while the rest of the IF2_mt_ protein was kept under strong harmonic restraints. The alternate model with altered orientation of the α-helix formed by residues 446 to 460 was generated by manually positioning that α-helix and the *ab initio* I-TASSER insert sequence region model, and then connecting the linker regions to the rest of the structure using LOOPY and optimizing the structure as mentioned above.

### Flexible fitting into cryo-EM maps

Since two of the cryo-EM maps used in this study do not have any density corresponding to IF1, the insert region was removed from the IF2_mt_ model and the resulting model was flexibly fit in the cryo-EM density maps using MDFF [41], [42]. To avoid over-fitting of quasi-atomic models to the lower resolution cryo-EM density maps, the MDFF protocol was optimized to choose the minimal number of optimization steps that would give the optimal correlation coefficient. The procedure used for choosing an MDFF protocol for IF2_mt_ models is illustrated in **Figure 4**. Six different MDFF protocols with varying number of optimization steps and different initial random velocities were used: (1) 5000 dynamics steps, 700 minimization steps; (2) 10000 dynamics steps, 1200 minimization steps; (3) 20000 dynamics steps, 2200 minimization steps; (4) 50000 dynamics steps, 5200 minimization steps; (5) 100000 dynamics steps, 10200 minimization steps; and (6) 150000 dynamics steps, 15200 minimization steps. The scaling factor specified by the gscale variable was set to 0.3 for the dynamics and first 200 minimization steps, and then set to 1.0 for the subsequent minimization steps. The correlation coefficient was found to plateau after MDFF protocol 5, which yielded an optimal fit with the least number of optimization steps. MDFF protocol 6 was not used since it could have caused over-fitting while yielding a very small further improvement in correlation coefficient as compared to MDFF protocol 5.

### Optimization of IF2_mt_ models based on IF2 density maps

The manually oriented 49 aa insert was reconnected individually to the two flexibly fit IF2_mt_ models using the program LOOPY [53] with the CHARMM22 protein parameters [54]. These two models were then subjected to the same optimization procedure using the program CHARMM [45] as mentioned above, involving multiple rounds of SD and ABNR minimizations and constrained room temperature langevin dynamics. To obtain the final complete optimized IF2_mt_ models, the insert and its neighboring regions (residues 464–518) in both models were subjected to restrained minimization and dynamics under center-of-mass restraints to keep the original location of the insert, while allowing its internal structure to adjust to the environment, and keeping the rest of IF2_mt_ under strong harmonic constraints.
